# A Metropolitan Police Hospital

**Published:** 1912-04-20

**Authors:** 


### A METROPOLITAN POLICE HOSPITAL.

In a recent letter to the Press a lady, who evidently takes a great interest in the welfare of the Metropolitan Police, pleads earnestly for the immediate establishment of a hospital devoted to the needs of sick or injured constables. Sympathising most fully with her solicitude for the proper care of the members of "the force" when ill, it may nevertheless be advisable to suspend judgment until every side of the question has been carefully considered. Immediate action in such a matter too often means frittered energy and unsatisfactory results.

It appears from Mrs. Cook's arguments that the City of London Police have a small hospital of thirty-two beds at the disposal of such of them as may need medical treatment. This hospital is staffed by a matron and two nurses, and is visited daily by the police surgeon. It contains also a consulting room and dispensary; by whom it is maintained is not stated. Now it is apparent from these *data* that the hospital is as a rule not full, probably not half full; or else that many of the cases received are trivial and could just as well be dealt with in the patients' own homes. It is also evident that no resident medical officer is appointed; and since the hospital serves a small area and is doubtless within easy reach of the surgeon, this is not a drawback. We may further assume that major surgery is not attempted, since there is no mention of an operating theatre; or else that it is carried on under great difficulties. All these considerations become germane when we contemplate the proposal to establish for the Metropolitan Police also a hospital for their own especial usage. If thirty-two beds are necessary (which we question) for a force of 1,000 men, then 640 will be required for a force of 20,000. Even if this estimate be halved, we are left with a 320-bed hospital, the expenses of erection and maintenance of which would be very great. The medical profession could scarcely be expected to staff it free of expense to the community; a resident staff as well as a visiting staff of physicians and surgeons would be needed, and district surgeons would still be required. However central in position such a hospital might be, it would of necessity be many miles from the homes of most of its patients.

Is such a hospital necessary? Mrs. Cook says so, and she has worked for many years among the homes of the Metropolitan Police. With her contentions about the value of rest, and quiet, and fresh air, and skilled nursing during illness everyone must agree; but she writes as if these are unobtainable for policemen who are ill. In their own quarters this may sometimes be the case; but the hospitals are only too glad to accept and care for policemen who are seriously ill, and many do in fact receive treatment therein. The chief surgeon is unwearied in arranging for such cases as are reported to him by the district surgeons being admitted into hospitals; and has taken hundreds of constables into his own wards for surgical treatment. The present system is economical and works well; it may in details be capable of improvement, but to represent it as a hopeless failure is an entire exaggeration, and the assertion that there is "practically no provision" for the men "being properly nursed and cared for" is to ignore the voluntary hospital system of London as if it did not exist. Whether reliance upon that system, and the acceptance of public charity thus entailed, is a proper way of meeting the needs of our police is another, and perhaps a debatable, matter. But at least there is time for discussing it calmly and without heat.

